# Combination effect of ultrasound-guided superior cervical ganglion block and standard triptan for migraine attacks

**DOI:** 10.22514/jofph.2025.038

**Published:** 2025-06-12

**Authors:** Hong Yue, Pengchao Li, Li Yuan, Guang Feng, Liangliang He

**Affiliations:** ^1^Department of Pain Management, Peking University Shougang Hospital, 100144 Beijing, China; ^2^Department of Orthopedic Surgery, Beijing Huguosi TCM Hospital, affiliated with Beijing University of Chinese Medicine, 100035 Beijing, China; ^3^Department of Acupuncture and Encephalopathy, Beijing Huguosi TCM Hospital, affiliated with Beijing University of Chinese Medicine, 100035 Beijing, China; ^4^Department of Reparative and Reconstructive Surgery, Peking University Shougang Hospital, 100144 Beijing, China; ^5^Department of Pain Management, Xuanwu Hospital, Capital Medical University, 100053 Beijing, China

**Keywords:** Superior cervical ganglion, Nerve block, Ultrasound guidance, Headache relief, Monthly migraine days, Migraine disability

## Abstract

**Background**: To examine whether the combination of ultrasound (US)-guided superior cervical ganglion (SCG) block with standard triptans has a superior relief of headache as compared to triptans alone for acute migraine. **Methods**: The retrospective-prospective cohort study enrolled patients presenting with acute migraine. The records of 243 cases receiving standard triptans and an adjunctive US-guided SCG were reviewed, while 230 cases were prospectively enrolled to receive triptan alone for control after age and sex matching in a 1:1.2 ratio. The primary endpoint was sustained headache relief and complete freedom from pain within post-procedural 24 hours. Secondary outcomes included headache relief and freedom from pain at 2 hours, monthly migraine days (MMDs), migraine disability assessment (MIDAS) scores, migraine-specific quality of life questionnaire (MSQ) scores, need for rescue analgesics use and adverse events. **Results**: Sustained headache relief was observed in 69.5% of cases in the SCG cohort, yielding a superior outcome increased by 21.7% (95% confidence interval (CI): 13.1%, 30.4%) (*p* < 0.001). Overall, SCG block plus triptans had a better effect on headache relief and freedom from pain at post-procedural 2 hours, and sustained freedom from headache within 24 hours compared to triptans alone. At 1-month visit, SCG cohort also showed a greater improvement in MMDs (*p* < 0.01), MIDAS scores (*p *= 0.040) and MSQ scores (*p *= 0.036). No major adverse events were observed. **Conclusions**: There was a superior therapeutic effectiveness of US-guided SCG block in conjunction with standard triptans for better headache relief up to post-procedural 1 month in acute migraine. It had an adjunctive beneficial effect in functional ability, life quality and possible facilitation of avoiding more adverse events.

## 1. Introduction

Migraine is a common neurological disorder characterized by recurrent attacks of disabling headaches [1]. Global epidemiology shows that it accounts for 4.9% of global population ill health quantified in years lived with disability [2]. The peak prevalence is observed in 7.9% (95% CI: 4.4%, 12.2%) for women and 3.1% (95% CI: 1.5%, 5.2%) for men among 40- to 49-year-olds from China mainland [3]. It not only limits patients’ quality of life, impairs work and social activities but also places an enormous burden on the healthcare system [4]. The complex pathophysiology of migraine that is also part of its burden, involves both central and peripheral mechanisms, including peripheral and central sensitization, lack of habituation, thalamo-cortical dysrethmia, and hyper-excitability of the motor cortex [5]. According to the classical neurovascular hypothesis, vasoactive neuropeptides, covering calcitonin gene-related peptide (CGRP), neurokinin A and substance *p* are released when the trigeminal sensory nerves are activated, and take part in vasodilation and dural plasma extravasation. The flow of nociceptive signals along the trigeminovascular pathway converges on the trigeminal nucleus caudalis and higher corticocerebral pain center of the brain to generate migraine pain [6]. The International Headache Society (IHS) global practice guidelines recommend non-steroidal anti-inflammatory drugs (NSAIDs) for mild attacks, and triptans for moderate to severe acute migraine. Despite aggressive pharmacological interventions, many patients continue to experience recurrent moderate to severe migraine pain [7]. The cervical sympathetic nerve blockade has been evaluated as a result of the need for innovative treatments to decrease the burden of migraines by reducing neurogenic dural vasodilation for pain alleviation [8]. It is supported by considerable evidence from autonomic manifestations, such as nausea, vomiting, diarrhea, cutaneous vasoconstriction, vasodilation, piloerection, diaphoresis, photophobia and abnormal pupillary reaction during acute migraine attacks [9]. Furthermore, migraineurs tend to have increased sympathetic activity during the ictal period [10]. The sympathetic fibers originating from the ipsilateral superior cervical ganglion (SCG) and the dense plexus around the meningeal artery and dural sinuses, modulate the vasomotor function [11]. Therefore, SCG blockade might be considered as a promising intervention for migraine patients with poor drug tolerability.

To our knowledge, SCG blockade has been reported for headaches or chronic facial pain, which is limited with very small samples [3]. The current study aimed to evaluate the accuracy, effectiveness and safety of a combined administration of ultrasound (US)-guided SCG block with standard triptans for the treatment of acute migraine in a large sample. We hypothesized that undergoing SCG block in conjunction with triptans would contribute to a better relief in headache as opposed to triptans alone.

## 2. Methods

### 2.1 Study design and participant selection

The protocol was approved by the institutional Ethics Examining Committee of Human Research (XW-KY-202417), and the study was conducted following the principles of the Declaration of Helsinki. The study was conducted at our pain center in accordance, results of which are reported in accordance with the Strengthening the Reporting of Observational Studies in Epidemiology (STROBE) guidelines [12].

This study comprised a retrospective phase to identify patients undergoing US-guided SCG block in combination with the standard first line medical treatment using triptans based on the Chinese guidelines for acute management of migraine between 01 January 2023 and 31 January 2024 [13]. All patients provided consent for using SCG block under US guidance and processing their medical data prior to treatment. Data was retrieved from electronic medical records (EMRs) and a prospective database based on our clinical protocol for migraine. From 01 February to 31 September 2024, patients with acute migraine attacks were prospectively enrolled in control cohort to receive the same standard triptans as the SCG cohort after age and sex matching in a 1:1.2 ratio (Fig. 1). Informed consent was obtained from all cases who agreed to participate in the research. Data collection was performed at the time of enrollment in our pain clinic, and at an interval of 2-hour, 24-hour and 1-month by two investigators through telephone interviews on pain free days using the same follow-up protocol as the SCG group.

**Fig. 1.** The flow chart of patient enrollment. US: ultrasound; SCG: superior cervical ganglion.

Inclusion criteria were as follows: (1) diagnosis of migraine consistent with the International Classification of Headache Disorders, 3rd edition (ICHD-3) [14]; (2) history of migraine greater than 1 year; (3) an acute attack lasting about 4 to 72 hours if untreated (4) an attack of moderate or severe intensity; (5) aged 18–50 years old. We excluded other headaches consequent for organic disorders, coagulation disorders, allergy to contrast medium, confounding chronic pain requiring regular analgesia, psychiatric illness, pregnancy/lactation, taking prophylactic medication. We also excluded individuals converting to other treatments during 1-month follow-up and those with incomplete data. Women who had migraine attacks that occurred only during the 5-day perimenstrual window, defined as pure menstrual migraine by ICHD-3, as at least two of three periods were also excluded [15].

Zolmitriptan tablets were provided at a dosage of 2.5 mg, and a second dose was available if symptoms persisted after 2 hours. However, the maximum dose could not exceed 15 mg within 24 hours. According to our routine protocol for headache, celecoxib tablets (200 mg, up to 2 times daily) were permitted as rescue analgesics if visual analogue scale (VAS) pain score was ≥4, while Oxycodone & Acetaminophen (5 mg: 325 mg tablets, up to 4 times daily) were available when VAS score was ≥7.

### 2.2 US-guided SCG block procedure

All procedures were performed as part of routine clinical practice by four pain doctors, who had a minimum of 5-year experience in the interventional techniques using US guidance for the treatment of headache.

Patients were placed in a supine position with the neck in a slight extension in operating room. A high-frequency US probe (6–13 MHz) was firstly placed with a transverse orientation next to the midline on the ipsilateral side of lower neck. It was moved up or down to identify the anatomic structures including the trachea, esophagus and thyroid gland (Fig. 2a). The transducer was further moved laterally to recognize the carotid sheath, which lied between the longus colli muscle (LCM) and sternocleidomastoid within the transducer range (Fig. 2b). Color doppler mode was applied to confirm the opposite blood flow between jugular vein and carotid artery. The carotid vein could be compressed by the excessive pressure on the skin through transducer, while the carotid artery could not. After the carotid artery was identified, the probe was gradually advanced to the cephalic direction along the carotid artery until the carotid bifurcation was obtained. The targeted SCG was located just posterior to the carotid bifurcation and anterior to the LCM in front of the 2nd to 3th cervical transverse process (TP) (Fig. 2c). Thereafter, the needle was inserted at a point 1 cm lateral to the linear probe and advanced towards the targeted SCG using the in-plane technique under real-time guidance (Fig. 2d). The precise needle tip position was verified by anteroposterior (AP) and lateral fluoroscopy by injecting 0.3 mL contrast medium to observe its diffusion range (Fig. 2e,f). After negative aspiration, 0.5 mL of 1% lidocaine was infiltrated into the fascial plane to block the SCG as the experimental dose. Patients were routinely monitored with electrocardiography, blood pressure and oxyhemoglobin saturation, as well as evaluated for signs of Horner’s syndrome every 5 min for a total of 20 min. Accordingly, patients were treated with an injection of 2% lidocaine (50 mg) and 5mg: 2 mg/mL betamethasone (0.5 mL) diluted with normal saline to 5 mL.

**Fig. 2.** US-guided SCG block with FL verification. (a) Midline structures of cervical region including trachea, esophagus and throid gland on the transverse US imaging; (b) The right carotid sheath and its’ surrounding structures in the lower cervical area; (c) The carotid bifurcation lying on the surface of LCM using dopplor model; (d) A needle (white arrows) was visualized from the lateral direction toward the targeted SCG between carotid bifuraction and LCM at the third cervical segment; (e) The anteroposterior view of FL confirmed the precise needle placement and contrast dispersion along cervical vertebras; (f) The lateral view of FL showed the needle tip locating at the third cervical vertebra and the contrast distribution covering the upper cervical segments. LCM: longus capitis muscle; OEC: orthopedic equipment company.

### 2.3 Outcomes measurement

A 11-point numeric rating scale (NRS) ranging from 0 to 10 was used to assess the severity of pain associated with acute migraine (0 = no pain; 1–3 = mild; 4–6 = moderate; 7–10 = severe) [16]. Headache relief was predefined as pain decreased from moderate or severe intensity to the levels of none or mild. Freedom from pain was defined as headache completely relieved to none from moderate or severe level. Monthly migraine days (MMDs) was the change from baseline in the number of days with a migraine attack over 1 month after two treatment modalities. The days of menstruation occurring were asked to be indicated for women patients. The migraine disability assessment (MIDAS) was employed to estimate the headache-related disability, which was composed of five questions concentrating on disability in three domains including school or paid work, household chores and family, social or leisure activities, as well as the other two questions focusing on the frequency and severity of migraine. The total score was categorized into four disability grades: 0–5 indicating minimal or infrequent disability; 6–10 indicating mild disability; 11–20 indicating moderate disability; ≥21 indicating severe disability [17]. The migraine-specific quality of life questionnaire (MSQ) version 2.1 was recorded to measure patients’ quality of life (QoL), which comprised 14-item questionnaires measuring QoL in three domains such as role function-restrictive (RFR), role function-preventive (RFP) and emotional function (EF). The total score summed all item scores and rescaled from 0–100, with a higher score indicating a better QoL [18]. Adverse events were also recorded. The primary endpoint was sustained headache relief at post-procedural 24 hours. Secondary outcomes included headache relief and freedom from pain at 2 hours, sustained freedom from pain at 24 hours, MMDs, MIDAS scores, MSQ scores, rescue analgesics and adverse events.

### 2.4 Statistical analysis

Statistics were processed with SPSS software version 22.0 (SPSS Inc, Chicago, IL, USA). Significant level was defined with *p *< 0.05. All data were checked for normality by Kolmogorov-Smirnov Z test. Nominal distribution data were recorded as mean ± standard deviation (SD) and compared by the Student *t* test, while non-normally distributed data were expressed as median ± inter quartile range (IQR) and compared using Mann-Whitney U test. Categorical data was presented as percentage and compared with Chi-squared test. To compare repeated measurement data over time, the repeated measures mixed-design analysis of variance (ANOVA) was used in-between cohorts after testing and correcting for sphericity, taking the baseline value as a covariate. *Post-hoc* comparison was conducted within cohort at an adjusted significance level of 0.05/3 = 0.017. Missing data on outcomes were excluded.

## 3. Results

Data from 243 cases were included in the SCG cohort for analysis after reviewing records from 301 patients with acute migraine. In addition, a total of 292 patients admitted for a migraine attack were prospectively screened for the control cohort after the performance of a 1:1.2 matching on age and gender. Of these, 24 cases not meeting inclusion criteria, 10 cases meeting exclusion criteria, 19 cases converting to other treatment and 9 cases lost to follow-up were excluded (Fig. 1). There were no significant differences between the two cohorts regarding demographic data and clinical characteristics of patients at baseline (Table 1).

**Table 1.** Demographic and clinical characteristics of participants at baseline.

Variables		SCG cohort (N = 243)	Control cohort (N = 230)	*p*
Age (yr) (mean ± SD)		45.07 ± 7.51	46.03 ± 8.29	0.640
Female sex, n (%)		152 (62.6%)	149 (64.8%)	0.633
BMI (kg/m^2^)		23.62 ± 2.53	24.58 ± 3.12	0.957
Diagnosis, n (%)				
	Migraine with aura	55 (22.6%)	46 (20.0%)	0.502
	Migraine without aura	188 (77.4%)	184 (80.0%)	
Duration of migraine (yr)		12.04 ± 8.98	11.76 ± 6.64	0.939
Affected side, n (%)				
	Left	101 (41.6%)	97 (42.2%)	0.973
	Right	87 (35.8%)	83 (36.1%)	
	Bilateral	55 (22.6%)	50 (21.7%)	
Headache attack duration, (h)		93.84 ± 17.09	90.42 ± 12.02	0.467
Acute headache medication, n (%)				
	None	41 (16.9%)	37 (16.1%)	0.970
	Migraine specific	165 (67.9%)	157 (68.3%)	
	Non-migraine specific	37 (15.2%)	36 (15.7%)	
Prior preventive treatment attempts, n (%)				
	None	137 (56.4%)	129 (56.1%)	0.657
	1 failed in past 1 yr	66 (27.2%)	69 (30.0%)	
	≥2 failed in past 1 yr	40 (16.5%)	32 (13.9%)	
NRS scores (median ± IQR)		6 (4, 8)	7 (5, 10)	0.698
Number of comorbidities		2.27 ± 1.53	2.60 ± 1.55	0.166

*SCG: superior cervical ganglion; BMI: body mass index; SD: standard deviation; NRS: numeric rating scale; IQR: interquartile range.*

Detailed efficacy results were revealed in Table 2. The SCG cohort reported sustained headache relief at post-procedural 2 hours in 90.5% of cases, while triptans alone yielded a slightly worse outcome decreased by 16.6% (95% CI: 9.9%, 23.4%) (*p* < 0.001). 69.5% of patients in the SCG cohort had freedom from pain within 2 hours, compared with 47.8% for the control cohort (mean difference (MD) = 21.7% (95% CI: 13.1%, 30.4%), *p *< 0.001). At 24 hours following treatment, sustained headache relief was observed in 73.3% of cases in the SCG cohort, compared with 53.0% in the control cohort, which was statistically significant with a superior MD of 20.2% (95% CI: 11.7%, 28.7%) and rate ratio (RR) equaling to (2.424 (95% CI: 1.651, 3.560)) (*p* < 0.001). Overall, SCG block plus triptan had a better effect on sustained freedom from headache within 24 hours, whereas triptan alone was associated with a less favorable outcome with RR of 2.503 (95% CI: 1.728, 3.626) (64.2% *vs.* 41.7%, *p* < 0.001). Table 2 also showed that a majority of patients reported at least moderate disability in both study cohorts at baseline (73.3% in SCG and 64.8% in control). At 1-month visit, the percentage of individuals with at least moderate disability was respectively reduced to 9.9% in the SCG cohort and 32.6% in the control cohort, indicating that the SCG cohort was associated with significantly lower incidence of moderate to severe disability in comparison with the control cohort. At 1-month visit, the mean of MMDs was 3.33 ± 1.44 in the SCG cohort and 5.65 ± 1.75 in the control cohort, which represented a decrease from the baseline of 10.97 ± 2.95 and 9.07 ± 4.80, respectively. However, the change from baseline to 1-month after treatment was statistically significant in the SCG cohort, if compared with the control cohort (*p* < 0.001). Although quantificational changes in migraine-specific quality of life questionnaire (MSQ) scores at 1-month visit were significantly higher than their baseline value in both cohorts, there was also a significantly greater improvement in MSQ scores in SCG block plus triptan compared with triptan alone at 1 months (78.54 ± 7.10 *vs.* 68.52 ± 11.35, *p *= 0.036).

**Table 2.** Primary and key secondary efficacy analysis between SCG cohort and control cohort.

Outcome			SCG cohort (n = 243)	Control cohort (n = 230)	Difference in rate (95% CI)	Rate ratio (95% CI)	χ^2^/*t* value	*p*
Pain relief, n (%)								
	2-hour headache relief		220 (90.5%)	170 (73.9%)	16.6% (9.9%, 23.4%)	3.376 (2.006, 5.682)	22.564	<0.001
	2-hour freedom from pain		169 (69.5%)	110 (47.8%)	21.7% (13.1%, 30.4%)	2.491 (1.710, 3.630)	23.044	<0.001
	24-hour sustained headache relief		178 (73.3%)	122 (53.0%)	20.2% (11.7%, 28.7%)	2.424 (1.651, 3.560)	20.800	<0.001
	24-hour sustained freedom from pain		156 (64.2%)	96 (41.7%)	22.5% (13.7%, 31.2%)	2.503 (1.728, 3.626)	23.942	<0.001
Monthly migraine days, (mean ± SD)								
	Baseline value		10.97 ± 2.95	9.07 ± 4.80			1.028	0.318
	1-month post-treatment		3.33 ± 1.44	5.65 ± 1.75			−8.061	<0.001
MIDAS, n (%)								
	Baseline value	Ⅰ	28 (11.5%)	37 (13.9%)			4.377	0.224
		Ⅱ	37 (15.2%)	49 (21.3%)				
		Ⅲ	69 (28.4%)	59 (23.5%)				
		Ⅳ	109 (44.9%)	85 (41.3%)				
	1-month post-treatment	Ⅰ	174 (71.6%)	108 (47.0%)			41.467	<0.001
		Ⅱ	45 (18.5%)	47 (20.4%)				
		Ⅲ	10 (4.1%)	33 (14.3%)				
		Ⅳ	14 (5.8%)	42 (18.3%)				
MSQ scores, (mean ± SD)								
	Baseline value		47.65 ± 4.64	47.33 ± 5.27			0.204	0.839
	1-month post-treatment		78.54 ± 7.10	68.52 ± 11.35			2.275	0.036

*SCG: superior cervical ganglion; SD: standard deviation; MIDAS: migraine disability assessment; MSQ: migraine-specific quality of life questionnaire; CI: confidence interval.*

Detailed results of rescue analgesics during the post-treatment times were reported in Fig. 3. The proportion of participants at 2 hours using analgesics was 5.2% and 9.2% for celecoxib and oxycodone-acetaminophen in the SCG block plus triptan cohort, compared with 11.8% and 27.5% of the triptan alone-treated participants (*p *= 0.063 and <0.001). 7.8% and 7.8% of patients required the above-mentioned analgesics for relief of headache in the SCG cohort, while 24.8% and 23.5% required the same analgesics within the control cohort at 24 hours visit (both *p *< 0.001). The proportion of participants in need of rescue celecoxib and oxycodone-acetaminophen at 1 month after treatment was 7.2% and 5.2% for SCG block plus triptan-treated cases, versus 17.6% and 19.6% for triptan only-treated patients (*p *= 0.009 and <0.001).

**Fig. 3.** Proportion of patients using rescue analgesics, and the mean dose changes of rescue analgesics based on the World Health Organization ladder across all time points during the follow-up period. SCG: superior cervical ganglion.

No serious complications including allergic reaction, arrhythmia, vascular injection, visible hematoma and local anesthetic intoxication were observed in our study. There were 4.2% of cases experiencing dizziness and somnolence in SCG cohort, as well as 5.0% in control cohort (*p *= 0.772). Intravascular contrast spread after the first attempt of needle placement was only observed in 1 patient in the cohort using US guidance. 5.1% of patients in the SCG cohort developed complications associated with steroid and local anesthetics injection including nausea, vomiting and facial flushing, however, all of them resolved within 30 min.

## 4. Discussion

Our primary result showed that the combination of both US-guided SCG block and conventional triptans for a migraine attack provided better headache pain relief up to post-procedural 1 month as opposed to triptans alone, with the absence of major adverse events including allergic reaction, arrhythmia, vascular injection, visible hematoma and local anesthetic intoxication.

Stellate ganglion block (SGB) referred to the blockade of cervical sympathetic chains/ganglia/fibers between the cervical and upper thoracic region, has been utilized to diagnose and treat multiple refractory pain related to head, face and upper extremities [19]. Stabilizing the sympathetic nerve dysfunction and suppressing the information of meningeal vascular wall are thought to be the mechanism to relief headache by SGB [20]. Previous trials reported the effectiveness and safety of US-guided SGB for the prophylactic treatment of migraine in the elderly. The mean number of headache days per month significantly reduced from 23.1 ± 5.5 to 10.9 ± 7.1 (*p* < 0.001), 12.7 ± 6.5 (*p* = 0.001) and 14.0 ± 6.8 days (*p* = 0.001) at the 1-, 2- and 3-month follow-ups, respectively [21]. The MIDAS scores were reduced to 7.0 ± 4.5 at 3-month follow-up [22]. Although SGB was an effective intervention for migraine, the SGB was absent in approximately 20% of the population [23]. Furthermore, it posed a risk of vertebral or thyroid arterial puncture leading to local anesthetic intoxication or hematoma even under the US guidance, because the puncture level was between the C6 and C7 segments [24].

SCG is part of the cervical sympathetic nervous system, which is situated on the LCM anterior to the TP of the 2nd to 3th cervical vertebrae [25]. Different from SGB, it is the uppermost part of the cervical sympathetic chain, which supplies densely sympathetic innervation around the cerebral vasculature. Therefore, SCG block may produce a more successful sympathetic blockade to the head than the conventional SGB, when the same dosage of local anesthetics (LA) is used [26]. Consequently, there is increasing evidence showing that the blockade of SCG is employed in cases of trigeminal neuralgia, post-herpetic neuralgia and chronic headache or facial pain [27]. Consistent with our hypothesis, US-guided SCG block had the capacity to alleviate pain in the acute management of migraine. With standard doses of zolmitriptan, only 73.9% and 47.8% of patients experienced relief and complete relieved headaches within 2 hours. Additionally, 24-hour sustained relief and complete freedom from pain were observed in 53.0% and 41.7% of the cases, which was also in accordance with a previous review reporting that standard triptans achieved acute migraine relief in 54% to 76% of patients, sustained freedom from pain in 18% to 50% within 2 hours, pain relief in 29% to 50% at 24 hours and sustained pain-free in 18% to 33% at 24 hours [28]. As compared to triptan alone, our results reported that the combination therapy of SCG block and triptans yielded significantly superior outcomes, because it provided significantly higher rates of pain relief and freedom from pain at 2 hours (90.5% and 69.5%) and sustained outcomes at 24 hours (73.3% and 64.2%) after treatment (Table 2). Consistent with our findings, Maeda A *et al*. [3] reported similar success in pain reduction for managing headaches and orofacial pain, where the maximum numeric rating scales (NRS) pain scores significantly reduced from 7.0 ± 0.7 to 4.5 ± 0.7 across 3 months after the application of SCG block procedures under US guidance (*p *= 0.014). Moreover, a previous open-label study reported that the incidence of attacks was decreased to 1 to 6 times per month, and freedom from attacks lasted 1/2 to 3/4 of a year and extended to 1 year or more in most of cases, if a repeated treatment with 6 to 8 blocks within the following years was continued [29]. Our results showed that the adjunctive SCG plus triptan treatment is superior to triptan alone because of the significantly lower monthly migraine days after blockade in this cohort (3.33 ± 1.44 *vs.* 5.65 ± 1.75 days). As a result, a possible hypothesis was deduced from the above-mentioned data, which highlighted the utilization of SCG blockade for migraine attacks. The infiltration with local anesthetic agent at the SCG would block the uppermost part of cervical sympathetic nerves passing through the SCG innervating the meningeal vessels. Therefore, dural blood flow would be significantly increased after the vasodilation of the meningeal vessels, resulting in a remission of subserve craniovascular pain that was an essential prerequisite to understanding the complex pathophysiology of migraine.

In addition, our results also reported a significantly lower proportion of patients with MIDAS scores 6 or higher over 1 month period after the SCG block procedure, as opposed to those in the control cohort (*p *= 0.040). Therefore, we presumed that SCG block was an effective option to not only relieve headache intensity but also improve MIDAS scores in patients with acute migraine, which aligns with previous study that proved the therapeutic role of cervical sympathetic nerve block in managing migraine attacks in 81 patients [30]. With regards to quality of life, the SCG cohort showed a greater improvement in MSQ scores than the control cohort (*p* = 0.036). Considering data from a previous randomized study, there could be a long-lasting clinical benefit of improved quality of life from the repetitive sympathetic nerve blocks [31]. When analyzing the usage of rescue analgesics, of particular note, there are indeed significant differences in the analgesic usage requirement between the systematic nerve block group and the control or placebo group based on previous researches for migraine [3, 22, 31]. In the present study, the SCG cohort reported significant decreases in the proportion of cases using rescue analgesics and the dosages of medication when comparing at 2 hours, 24 hours and 1month post-treatment, thus corroborating the previous studies.

Ultrasound-guided technique by color-doppler modality as applicable offers clinical advantages in the performance of nerve blocks in terms of portable, real-time imaging and radiation-free [32]. This technique has rendered SCG block an effective and safe intervention as reported by a few case reports [33]. It has been speculated that the US technique is associated with lower injectate volumes and fewer complications, which coincides with the present study. A total of 2 mL injectate was found to be sufficient for the presence of a Horner’s syndrome without severe events in the present study. The incidence of minor adverse events related to the US-guided SCG block procedure was 9.3%, all of which were transient, with dizziness and facial flushing being the most common.

This study had several limitations. First, investigators responsible for outcome assessment failed to ensure blindness due to the retrospective-prospective nature of the study. Second, since data collection did not allow us to identify women who had both menstrual and non-menstrual attacks, we could not exclude the impact of menstrual cycle on migraine attacks. Third, we did not compare the effect of SCG block to alternative treatments, such as CGRP monoclonal antibodies or neuromodulation techniques. Fourth, given that there was no plan on examining the efficacy in subgroups of patients, we could not identify a specific patient subtype that might benefit more from SCG blockade. Fifth, patients were not entirely prevented from using analgesics, which might yield confounding bias. The lack of long-term follow-up on cumulative benefits from repeated SCG blocks was also a limitation in this study, therefore, a well-designed, randomized, controlled study with an extended follow-up period to assess the results of this analysis is recommended in the future.

## 5. Conclusions

In conclusion, a combination of both US-guided SCG block and standard triptans appeared to yield superior relief of headache up to post-procedural 1 month, when compared with triptans alone. Notably, this technique had an adjunctive beneficial effect in functional ability and quality of life, and avoided a few minor adverse events related to the conventional stellate ganglion block (SGB).
